# 543 Hospital Acquired Burn Cellulitis in a Single ABA Verified Burn Center

**DOI:** 10.1093/jbcr/iraf019.172

**Published:** 2025-04-01

**Authors:** Sylvonne Layne, Kaitlyn Libraro, Abraham Houng

**Affiliations:** Weill Cornell Medical College; Weill Cornell Medical College; New York Presbyterian Hospital - Weill Cornell

## Abstract

**Introduction:**

Cellulitis is a known complication in burn injuries. It is one of the leading infections in burn patients. Risk factors for cellulitis include delay in treatment, history of diabetes and burns that require surgery. The rate of burn cellulitis has been published in numerous studies. However, hospital acquired burn cellulitis is not well known. Through our burn center’s quality improvement program, we examined our institution’s hospital acquired burn cellulitis rate in a given year.

**Methods:**

Data from weekly quality improvement meeting was used to capture burn patients with cellulitis. Hospital acquired burn cellulitis was defined as patients admitted without cellulitis, but developed cellulitis around the burn wound after 24 hours of admission. Study time ranged from 1/1/2023 to 12/31/2023. Data abstracted included demographics, medical history, injury details and hospital course.

**Results:**

In 2023, we had 533 admissions to the burn unit. 23 patients had admission diagnosis of cellulitis and thus were excluded from the study. Of the 510 patients, 12 were diagnosed with cellulitis with a rate of 2.4%. Average hospital day of cellulitis diagnosis was 4.45 days (SD 1.86). Of the 12 patients, 8 were burns of lower extremities, while 4 were on upper extremities. In terms of mechanism, 4 were oil scald, 3 were flame, 3 were hot water scald, and 2 were abrasion. Burn surgery was recommended in 11 of the 12 patients with cellulitis. 2 patients were diabetics. All 12 patients sought treatment within 12 hours of their injury.

**Conclusions:**

Burn wound cellulitis is a common infection in burn patients. We provided data on hospital acquired burn wound cellulitis in our institution to better understand the disease process. Based on this preliminary data, we do not see a pattern or trend. There were no specific risk factors for hospital acquired cellulitis that we could easily identify. We will continue to trend data with our quality improvement team to increase the sample size and use univariate analysis to identify potential risk factors.

**Applicability of Research to Practice:**

Directly applicable

**Funding for the Study:**

N/A

